# Linking Clinical and Environmental Multidrug Resistance Plasmids Captured from the Tama River Flowing Through the Tokyo Megalopolis

**DOI:** 10.3390/antibiotics15030241

**Published:** 2026-02-25

**Authors:** Rin Yamazaki, Maho Tokuda, Singh Shweta, Koichiro Nakamichi, Ryota Moriuchi, Hideo Dohra, Hiroyuki Futamata, Kazuhide Kimbara, Masaki Shintani

**Affiliations:** 1 Graduate School of Integrated Science and Technology, Shizuoka University, Shizuoka 432-8561, Japan; yamazaki.rin.20@shizuoka.ac.jp (R.Y.); tokuda-maho@tmu.ac.jp (M.T.); nakamichi.koichiro.16@shizuoka.ac.jp (K.N.); dora.hideo@shizuoka.ac.jp (H.D.); futamata.hiroyuki@shizuoka.ac.jp (H.F.); kimbara.kazuhide@shizuoka.ac.jp (K.K.); 2 Faculty of Science, Tokyo Metropolitan University, Tokyo 192-0397, Japan; 3 Graduate School of Science and Technology, Shizuoka University, Shizuoka 432-8561, Japan; singh.shweta.21@shizuoka.ac.jp; 4 Shizuoka Instrumental Analysis Center, Shizuoka University, Shizuoka 422-8529, Japan; moriuchi.ryota@shizuoka.ac.jp; 5 Research Institute of Green Science and Technology, Shizuoka University, Shizuoka 422-8529, Japan; 6 Japan Collection of Microorganisms, RIKEN BioResource Research Center, Ibaraki 305-0074, Japan

**Keywords:** plasmids, transposon, class 1 integron, IS, TIME

## Abstract

**Background:** Plasmid-mediated horizontal transfer of antimicrobial resistance genes (ARGs) is a major driver of resistance dissemination across clinical and environmental settings. Urban rivers flowing through densely populated megacities represent critical interfaces where human-associated and environmental microbiomes intersect; however, the genetic structures and functional characteristics of resistance plasmids circulating in such environments remain insufficiently resolved. **Methods:** In this study, we conducted detailed genomic and phenotypic analyses of 11 ARG-bearing plasmids previously captured from the Tama River, an urban river flowing through the Tokyo megalopolis. These plasmids belonged to IncN, IncU, IncQ2γ, IncC, and IncPγ groups. Whole-plasmid sequencing, comparative genomic analyses, conjugation assays, and antimicrobial susceptibility testing were employed to characterize plasmid backbones, accessory resistance regions, mobile genetic elements, and conjugative transferability. **Results:** A total of 11 plasmids belonging to five major incompatibility groups (IncN, IncC, IncU+IncQ2γ, and IncP) were analyzed. These plasmids collectively encoded ARGs conferring resistance to five major antimicrobial classes, including aminoglycosides, β-lactams, tetracyclines, chloramphenicol, and mercury, and frequently harbored class 1 integrons, IS*CR1* elements, and Tn*3*-derived inverted-repeat miniature elements (TIME). Notably, two plasmids (IncN and IncC) exhibited high structural similarity to clinically reported plasmids from geographically distant regions, whereas multiple IncP plasmids and one multi-replicon IncU+IncQ2γ plasmid displayed accessory-region architectures characteristic of environmental plasmids and broad host-range transferability. Antibiotic susceptibility testing demonstrated that these plasmids substantially increased resistance levels in hosts. **Conclusions:** This study reveals that urban river environments can harbor both clinically related and environmentally unique multidrug resistance plasmids, shaped by diverse mobile genetic elements. By providing nucleotide-level structural and functional evidence, this study highlights urban rivers as potential ecological hubs linking clinical and environmental resistance plasmid pools and supports the importance of continued monitoring of resistance plasmids in megacity-associated river systems.

## 1. Introduction

The emergence and spread of antimicrobial-resistant bacteria poses a serious threat to global health. A particularly concerning mechanism behind this resistance is the spread of antimicrobial resistance genes (ARGs) via plasmids, one of the mobile genetic elements (MGEs), that can transfer between different bacterial species through conjugative transfer [1]. Plasmids carrying ARGs, known as resistance plasmids (R plasmids), are classified into incompatibility (Inc) groups based on the similarity of their replication systems. Many well-known R plasmids belong to groups such as IncC, IncF, IncN, IncL, among others, and IncC and IncN plasmids are capable of horizontal transfer across a wide range of bacterial hosts [2,3]. These plasmids play a central role in the dissemination of resistance traits not only in clinical settings but increasingly in the environment [1].

Recent studies have reported the presence of multidrug-resistant bacteria and ARGs in wastewater plants and urban rivers [4,5,6,7], as well as on agricultural products such as vegetables [8,9]. These findings suggest that ARG transfer is occurring in environments closely linked to daily human life. R plasmids originating from human clinical isolates have been well studied. However, such investigations in natural environments are still limited. Little is known about whether R plasmids are capable of propagating in environmental bacterial communities, or what kinds of bacteria are involved in such exchanges. Moreover, it is plausible that novel plasmids, distinct from those characterized in clinical strains, exist in natural environments and contribute to the emergence of resistance. It is therefore important to understand the linkage between R plasmids in clinical and natural environments.

The Tama River flows through the Tokyo megalopolis, one of the world’s most densely populated urban areas, where inputs from households, hospitals, industries, and wastewater treatment plants converge. This urban river therefore represents a critical human–environment interface in which clinical, anthropogenic, and environmental microbial communities can interact.

In our previous study, we collected 167 transconjugants from the Tama River using the exogenous plasmid capture method, revealing that diverse plasmids circulate in this urban river environment [10]. However, the genetic structures, MGEs, and antimicrobial resistance phenotypes of these plasmids remained largely unexplored. In the present study, we focused on 11 plasmids from this collection that were found to carry ARGs. These plasmids belonged to several incompatibility groups—IncN, IncU, IncQ2, IncC, and IncP—representing key vectors known to mediate multidrug resistance (MDR) in both clinical and environmental settings. We performed comprehensive genomic analyses of their backbone regions, accessory resistance regions, and associated MGEs, including integrons, insertion elements (ISs), transposons, and Tn*3*-derived inverted-repeat miniature elements (TIMEs) [11]. We additionally conducted conjugation assays and antimicrobial susceptibility testing to assess their transmissibility and resistance phenotypes. By comparing the plasmids with related sequences reported worldwide, we aimed to clarify the evolutionary connections between environmental and clinical plasmid pools and to better understand the role of urban rivers as reservoirs and dissemination points of MDR plasmids.

## 2. Results and Discussion

### 2.1. Various Plasmids Carrying ARGs Were Captured from the Tama River

**Figure 1:**
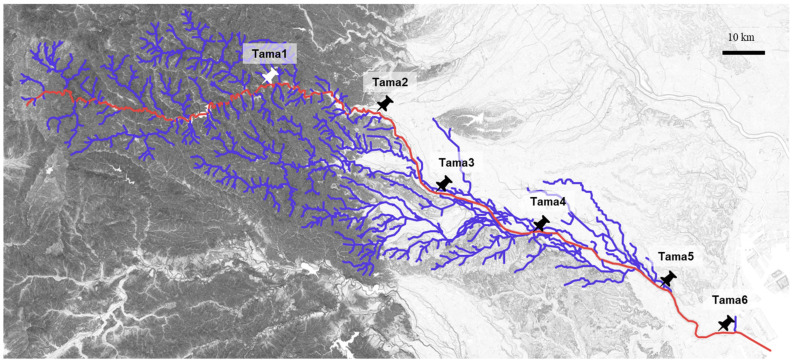
Locations of the sampling sites are shown on the map. The mainstream of the Tama River is shown in red, and its tributaries are shown in blue.

Our previous exogenous plasmid captures using microbial communities collected from six sampling sites along the Tama River (Tama1 to Tama6, Figure 1) yielded 167 transconjugants through biparental matings and triparental matings, most of which were previously analyzed [10]. In principle, biparental matings allow the capture of plasmids that both confer the targeted resistance to the hosts and are self-transmissible between different cells. In contrast, triparental matings enable the acquisition of self-transmissible plasmids that do not carry the marker resistance genes but can mobilize non-self-transmissible plasmids as helper plasmids. Here, we specifically examine 11 plasmids carrying ARGs that were captured from midstream or downstream sites of the Tama River (Tama4, Tama5, and Tama6, Figure 1 and Table 1). There were seven IncP plasmids, including IncPβ, IncPγ, IncPε, IncPι and IncPκ subgroups, one IncN plasmid, and one IncC plasmid (Table 1). One plasmid, pMNBM065-2, was estimated to be classified both under the IncU and IncQ groups (a multi-replicon). In addition, a PromA plasmid (pMNBM065-1) was identified in the same host as pMNBM065-2; however, as it did not carry any ARGs, it was excluded from further analysis (Table 1). The 11 plasmids harbored resistance genes against aminoglycosides, β-lactams, tetracyclines, mercury, and other agents. Hosts carrying these ARG-bearing plasmids exhibited resistance to the tested antibiotics, including tetracycline (Tc), gentamicin (Gm), ampicillin (Ap), kanamycin (Km), chloramphenicol (Cm), streptomycin (Sm), and erythromycin (Em), as summarized in Table 2.

In the following sections, we present detailed nucleotide sequence-level analyses and comparative genomic analyses of each individual plasmid and the mobile genetic elements associated with ARGs on these plasmids.

### 2.2. Multi-Replicon Plasmid pMNBM065-2

**Figure 2:**
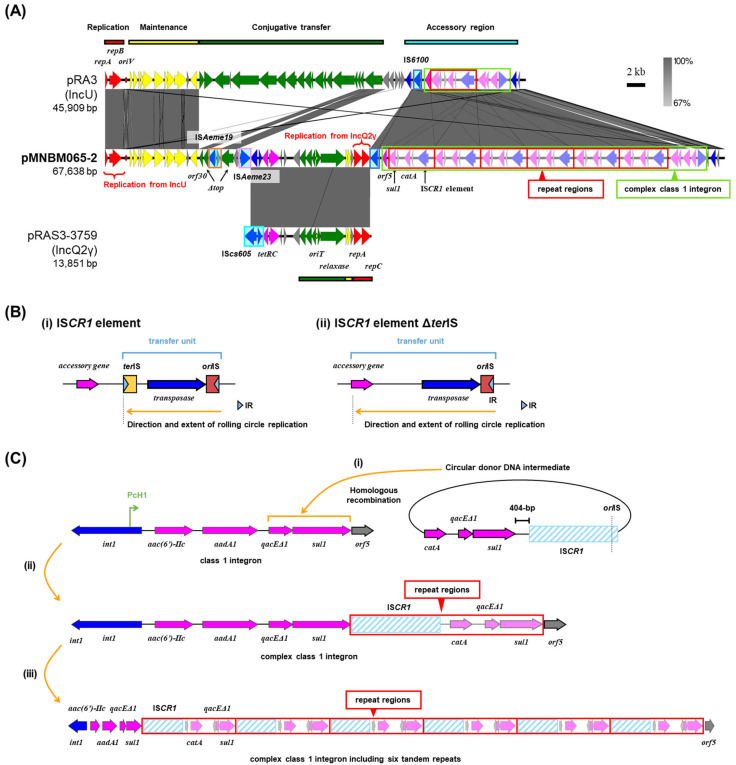
Alignment of pMNBM065-2, IncU plasmid pRA3, and IncQ2γ plasmid pRAS3-3759 (**A**). Coding DNA region, their directions, and their predicted functions are indicated as block arrows with colors, red for replication, yellow for maintenance, green for conjugative transfer, blue for genes related to mobile genetic element, pink for ARG, and gray for other genes. Insertion sequences (ISs) are shown as sky blue-filled boxes. Complex class 1 integron and repeat regions are boxed in light green and red, respectively. (**B**) The construction of IS*CR1* element (**i**) and IS*CR1* element Δ*ter*IS (**ii**). (**C**) The formation process of a complex class 1 integron. A circular donor DNA intermediate containing IS*CR1* element and non-cassette resistance genes was inserted in an original class 1 integron (**i**). A complex class 1 integron was formed (**ii**), followed by six tandemly arranged regions containing IS*CR1* element, *catA*, *qacEΔ1* and *sul1* (**iii**).

Plasmid pMNBM065-2 was captured downstream (Tama5) along with pMNBM065-1 (PromAγ) through biparental matings (Table 1). pMNBM065-2 was shown to be a multi-replicon plasmid possessing genes encoding replication initiation protein (RIP) of both IncU and IncQ2γ (Figure 2A). A comparison of the nucleotide sequences of pMNBM065-2 with pRA3, an archetype plasmid of IncU group [12,13] and pRAS3-3759, a member of IncQ2γ plasmid [14,15,16], showed that pMNBM065-2 contained one region homologous to maintenance and accessory regions of pRA3 (IncU) and another region highly similar to almost the entire sequence of pRAS3-3759 (IncQ2γ) (Figure 2A). This plasmid additionally had six tandem 5134 bp repeat regions, containing resistance genes for sulfonamide (*sul1*) and chloramphenicol (*catA*) and a putative gene encoding a transposase of IS*CR1,* a member of the IS*91* family (Table 1, Figure 2A).

IS*CR1* elements encode a transposase that catalyzes transposition through a rolling-circle mechanism [17,18,19]. They possess two imperfect terminal 8 bp inverted repeats, *ter*IS (the left end) and *ori*IS (the right end), each associated with characteristic dyad-symmetry sequences that serve as recognition sites for the transposase [20] (Figure 2B(i)). The *ori*IS region, located downstream of the transposase gene, is essential for initiating rolling-circle replication during transposition [18] (Figure 2B(i)). A notable feature of the IS*CR1* element is that a single copy of the element is sufficient to mobilize adjacent DNA sequences when the ~100 bp *ori*IS region is present [18] (Figure 2B(ii)). This property enables one-ended transposition, in which transposition proceeds from the *ori*IS end without requiring a second IS end (*ter*IS) [19] (Figure 2B(ii)).

Previous studies have shown that IS*CR1* elements are almost exclusively associated with class 1 integrons [21]. A genetic model has been proposed in which the IS*CR1* element is fused to the class 1 integron. The 3′-conserved segment (3′-CS) of the integron is formed by the fusion of the *sul1* and *qacEΔ1* genes [21]. In this structure, fusion of IS*CR1* with the 3′-CS results in deletion of the *ter*IS site, and the start codon of the IS*CR1* ORF is located 404 bp downstream of the stop codon of the *sul1* gene [20]. In addition, the gene *orf5*, which is typically found downstream of *qacEΔ1*-*sul1*, is absent [20]. The fusion of IS*CR1* to the 3′-CS, together with the absence of *ter*IS, allows the IS*CR1* element to undergo rolling-circle replication encompassing all or part of the class 1 integron. This process enables mobilization of the class 1 integron [20]. Mobilized class 1 integrons can subsequently acquire non-cassette resistance genes, and recombination with the 3′-CS of another class 1 integron leads to the formation of a complex class 1 integron [20] (see Figure 2C as follows).

In pMNBM065-2, we identified two sequence features relevant to IS*CR1*-mediated rearrangements. First, a highly conserved 22 bp sequence (5′-GTGGTTTATACTTCCTATACCC-3′) [21,22] was detected at the right end of the IS*CR1* element, which is consistent with an *ori*IS-like site (Figure 2C). Second, the stop codon of *sul1* and the start codon of the IS*CR1* ORF were separated by 404 bp (Figure 2C), a spacing previously reported for IS*CR1* elements fused to the 3′-CS of class 1 integrons. These observations support a plausible scenario in which IS*CR1* element, together with *catA* and *qacEΔ1*-*sul1*, generated a circular donor DNA intermediate via one-ended transposition, followed by recombination into the 3′-CS of the class 1 integron on pMNBM065-2 (Figure 2C(i)). If this scenario occurred, the architecture shown in Figure 2C(i) would be expected, namely a complex class 1 integron containing aminoglycoside resistance genes [*aac(6′)-IIc* and *aadA1*] plus the IS*CR1*-associated region (*qacEΔ1*-*sul1*-IS*CR1*-*catA*) with partial copies of *qacEΔ1* and *sul1* (Figure 2C(ii)). Reiteration of this recombination process could account for the presence of six tandem repeats of the region containing the IS*CR1* element, *catA*, and partial *qacEΔ1*-*sul1* sequences (Figure 2C(iii)). However, subsequent sequencing after cultivation indicated that this region was detected in only one or two copies, suggesting this repeat region may be unstable or subject to rearrangement under the cultivation condition used.

For the minimum inhibitory concentration (MIC) analysis, the plasmid pMNBM065-2 containing two copies of the repeat region was used. The MIC of the parental *E. coli* MG1655RGFP, which carries a single chromosomal *catA* gene inserted with a GFP gene by a mini-transposon [23], against chloramphenicol (Cm), determined by broth microdilution, was 250 µg/mL, while that of *E. coli* MG1655RGFP harboring pMNBM065-1 and pMNBM065-2 was 2000 µg/mL, representing an eightfold increase (this assay was conducted using two biological replicates with two technical replicates each). The pMNBM065-2 also carried tetracycline (*tetRC*) and conferred resistance to its host (Table 2). It should be noted that no known ARGs were detected on pMNBM065-1 (Table 1).

### 2.3. Mobilization of pMNBM065-2

Plasmid pMNBM065-2 possessed partial gene sets for conjugative transfer found in IncU plasmids, namely *orf30*-*top* genes [13], but the *top* gene was disrupted by the insertion of IS*Aeme19*, and additional putative relaxosome-related genes (MOB_P_ family gene) and *oriT* region (137 bp) found in IncQ2γ plasmids (Figure 2A). The co-resident plasmid pMNBM065-1 (PromAγ) is self-transmissible, which has MOB_P_ and MPF_T_ family genes. To assess whether the pMNBM065-2 could be mobilized by pMNBM065-1, filter mating assays with *E. coli* JM109(pUC19) were conducted. As a result, both plasmids were transferred to the recipient with a transfer frequency of 1.7 × 10^–2^ per donor (two independent experiments). Although IncU and IncQ2γ plasmids were mainly isolated from *Enterobacteriaceae*, the transfer range of the PromAγ plasmid is known to be broad [24]. Therefore, pMNBM065-2 is likely to transfer among different bacterial families beyond its native host range, thereby conferring antimicrobial resistance and enhancing the survival of recipient bacteria in the Tama River.

### 2.4. IncN Plasmid pMNBM072

**Figure 3:**
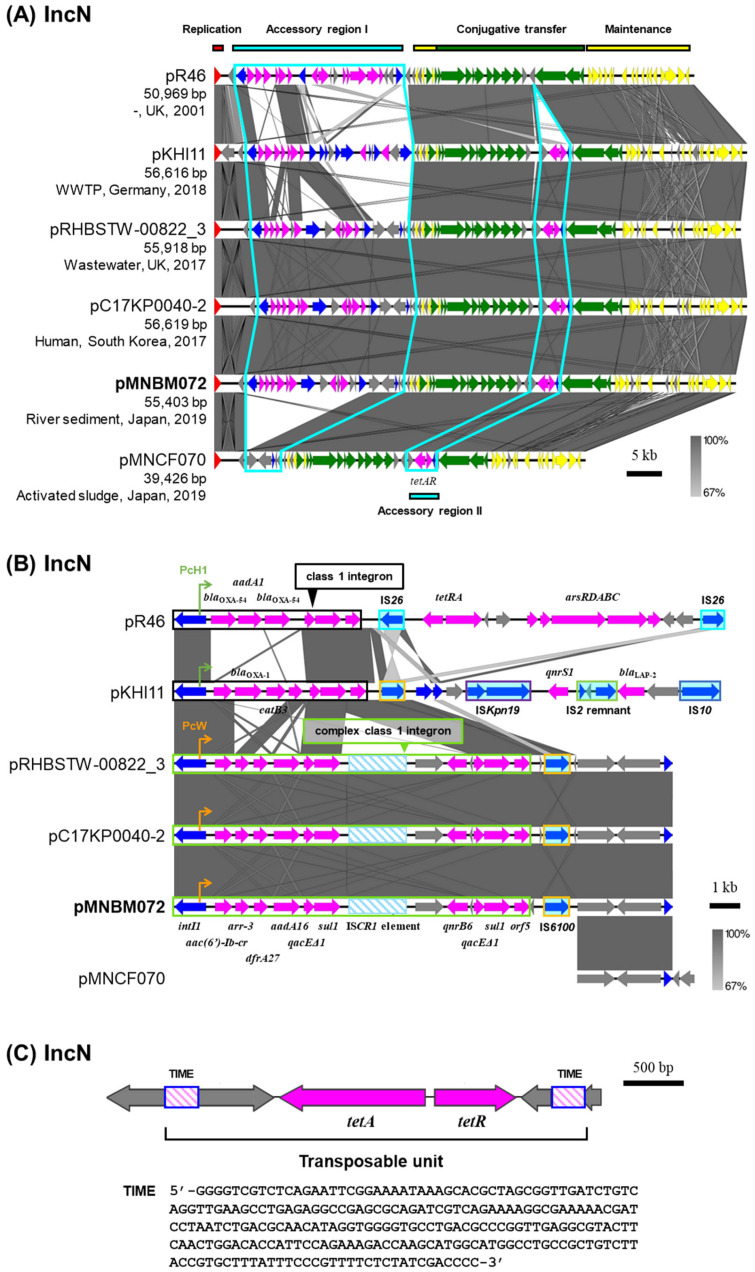
Alignment of IncN plasmids isolated from different sources (**A**) and the accessory region I including antimicrobial resistance genes and mobile genetic elements (**B**). Two regions inserted accessory genes are outlined with sky blue lines. Coding DNA region, their directions, and their predicted functions are indicated as block arrows with colors, red for replication, yellow for maintenance, green for conjugative transfer, blue for genes related to MGE, pink for ARG, and gray for other genes. IS are shown as sky blue-filled boxes. (**C**) The genetic structure of Tn*3*-derived inverted-repeat miniature elements (TIMEs) containing *tetAR* and its nucleotide sequences.

Plasmid pMNBM072 was captured downstream (Tama5) through biparental matings and classified under the IncN plasmid group (Table 1). IncN plasmids were detected in clinical and environmental sources worldwide, and their nucleotide sequences were highly conserved with the IncN archetype plasmid pR46 [25] (Figure 3A). Two accessory regions were identified, each probably inserted at specific regions, between the replication and conjugative transfer region (accessory region I) or downstream of the relaxase gene (accessory region II) [6]. pMNBM072 possessed a complex class 1 integron with gene cassettes *aac(6′)-Ib-cr*, *arr-3*, *dfrA27*, *aadA16*, *qacEΔ1*, *sul1*, IS*CR1* element, *qnrB6*, *qacEΔ1* and *sul1* in the accessory region I (Figure 3B). Among them, a single copy of *qacEΔ1*-*sul1*, IS*CR1* element and *qnrB6* were likely inserted through transposition and recombination mediated by the IS*CR1* element fused to the 3′-CS of the class 1 integron. In addition, the IS*6100* element was located within the same region and 25 bp inverted repeats (IRs) (5′-TGTCRTTTTCAGAAGACGRCTGCAC-3′) and 5 bp direct repeats (DRs) (5′-CTGTT-3′) were detected outside of the IS*6100* element and the class 1 integron. Consistent with the presence of these resistance genes, pMNBM072 conferred resistance to kanamycin, streptomycin and tetracycline on its host (Table 2).

Moreover, pMNBM072 and the IncN plasmids isolated from the United Kingdom and South Korea were highly similar in terms of both their backbones and accessory genes (Figure 3A,B). Additionally, pMNBM072 carried resistance genes for tetracycline (*tetAR*) in the accessory region II and conferred resistance on its host (Table 2). A region containing *tetAR* was flanked by 244 bp Tn*3*-derived inverted-repeat miniature elements (244 bp TIMEs) and 5 bp DR (5′-AGCAA-3′), which is a short and non-autonomous transposable element characterized by terminal inverted repeats but lacking the capacity to encode a transposase (Figure 3C) [11,26]. This region is likely mobilized by transposases provided in trans, thereby facilitating the dissemination of resistance genes.

### 2.5. IncC Plasmid pMNBL073

**Figure 4:**
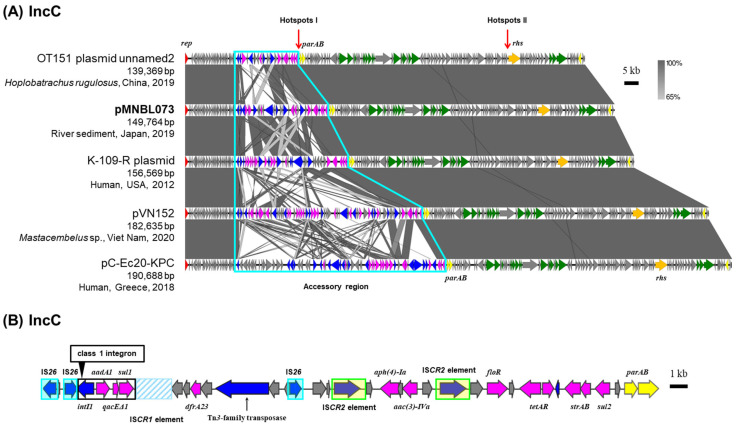
Alignment of IncC plasmids isolated from different sources (**A**), and the genetic structure of accessory region I of plasmid pMNBL073 (**B**). A region’s inserted accessory genes are outlined with sky blue lines. Coding DNA region, their directions, and their predicted functions are indicated as block arrows with colors: red for replication, yellow for maintenance, green for conjugative transfer, blue for genes related to MGE, pink for ARG, and gray for other genes.

Plasmid pMNBL073 was captured from midstream site (Tama4) through biparental matings and estimated to be classified under the IncC group based on PCR analyses (Table 1) [10]. Based on the full-length nucleotide sequences of RIP genes, pMNBL073 was a member of the IncC group. IncC plasmids have been isolated from a wide range of sources, including humans, animals, and natural environments. Their backbone sequences are highly similar (Figure 4A), and accessory regions are predominantly inserted at two specific regions’ ‘hotspots’ [27]. In pMNBL073 and its homologous plasmids, an accessory region was also identified upstream of *parA* gene, at one of the specific insertion regions (Figure 4A) [27]. A comparison of the nucleotide sequences of this accessory region showed that the antimicrobial resistance genes, transposons and IS present were diverse and complex (Figure 4A). The other ‘hotspot’ is upstream of the *rhs* gene. pMNBL073 carried genes conferring resistance to aminoglycosides [*aadA2*, *aac(3)-IVa*, *aph(4)-Ia* and *strAB*], sulfonamide (*sul1* and *sul2*), trimethoprim (*dfrA23*), chloramphenicol (*floR*) and tetracycline (*tetAR*) (Figure 4B). Among these resistance genes, *aadA2*, *qacEΔ1* and *sul1* are included in a class 1 integron (Figure 4B). pMNBL073 conferred resistance to gentamicin, chloramphenicol, and tetracycline on its host (Table 2).

### 2.6. IncP Plasmids

**Figure 5:**
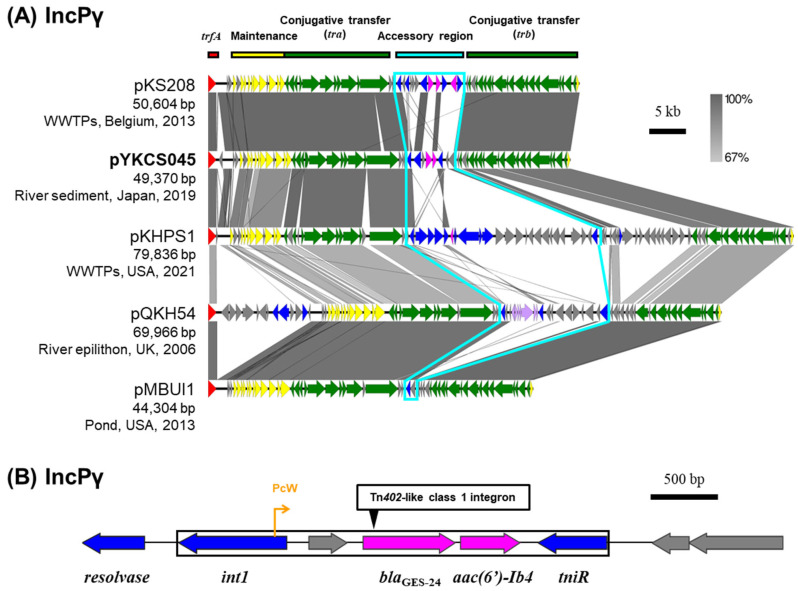
Alignment of IncPγ plasmids (**A**) and the genetic structure of Tn*402*-like class 1 integron of plasmid pYKCS045 (**B**). A region’s inserted accessory gene is outlined with sky blue lines. Coding DNA region, their directions, and their predicted functions are indicated as block arrows with colors: red for replication, yellow for maintenance, green for conjugative transfer, blue for genes related to MGE, pink for ARG, and gray for other genes.

Among the seven IncP plasmids captured from the Tama River, plasmids pYKCS045 (IncPγ), pMNBL056 (IncPε), and pYKBL037 (IncPι) carried class 1 integrons containing multiple ARGs (Table 1) [10]. pYKCS045 captured from the downstream site of the Tama River (Tama5) through triparental matings was predicted to be a IncPγ plasmid. Only four other IncPγ plasmids are registered in the plasmid database PLSDB [28,29,30], and they were isolated from natural environments such as rivers, wastewater treatment plants (WWTPs) and ponds [31,32]. A comparison of the nucleotide sequences of IncPγ plasmids showed that their core genes for replication, maintenance, and transfer were highly conserved (Figure 5A). The accessory regions including ARGs were inserted between *tra* gene sets and *trb* gene sets. Plasmid pYKCS045 carried the resistance genes for carbapenems (*bla*_GES-24_) and aminoglycosides [*aac(6′)-Ib4*] within a Tn*402*-like class 1 integron (Figure 5B) with 25 bp IRs (5′-TGTCRTTTTCAGAAGACGRYTGCAC-3′) and 5 bp DRs (5′-CCTAT-3′). By broth microdilution, the MIC of *Metapseudomonas resinovorans* CA10dm4RGFP harboring this plasmid against meropenem was 32 µg/mL.

### 2.7. Class 1 Integrons

**Figure 6:**
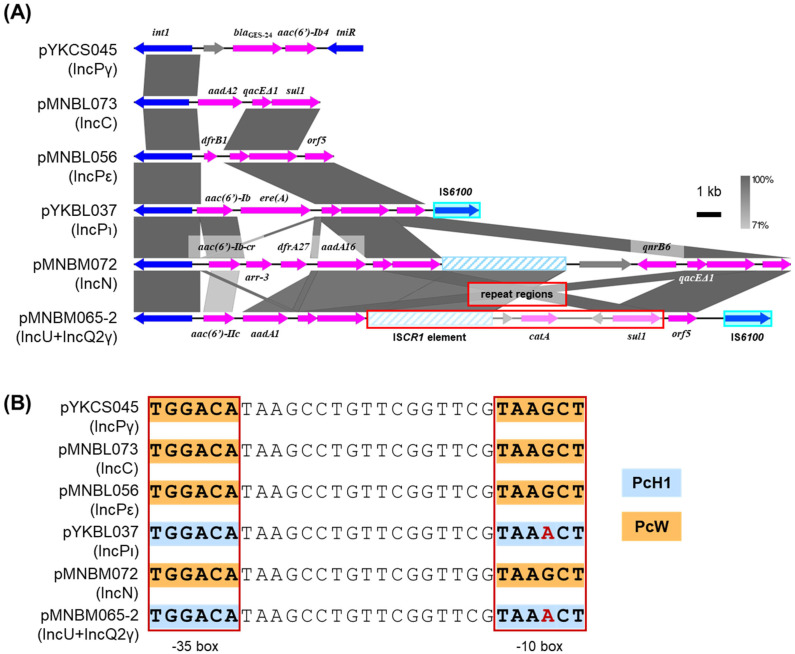
Comparisons of the genetic structure for the class 1 integrons found in different plasmids from the Tama River (**A**). The repeat region of plasmid pMNBM065-2 is shown as a single copy for clarity. Coding DNA regions, their directions, and their predicted functions are indicated as block arrows with colors: blue for genes related to mobile genetic element, pink for genes related to antimicrobial resistance genes, and gray for other genes. IS are shown as sky blue-filled boxes. (**B**) Comparisons of promoters for gene cassettes in the class 1 integrons. Red boxes show –35 and –10 regions of the gene cassette and +1 indicates transcription start point. The boxes with orange indicate PcW promoter, while those with light blue indicate PcH1 promoter.

Regardless of the Inc groups, class 1 integrons were found in six plasmids including pYKCS045 (IncPγ), pMNBL056 (IncPε), pYKBL037 (IncPι), pMNBM065-2 (IncU and IncQ2γ), pMNBM072 (IncN) and pMNBL073 (IncC). Plasmid pYKCS045 had Tn*402*-like class 1 integrons, which contained a part of transposase genes, *tniR*, *bla*_GES-24_ and *aac(6′)-Ib4* (Figure 6A). Plasmids pMNBL056 and pYKBL037 had a class 1 integron 3′ conserved segment (3′-CS) with three well-conserved genes, *qacEΔ1*-*sul1*-*orf5*, while pMNBL073 contained a similar integron with *aadA2* gene and 3′-CS, lacking an *orf5* gene (Figure 6A) [33]. In addition, the integron of pMNBL056 contained the gene cassette *dfrB1* encoding for trimethoprim resistance, whereas that of pYKBL037 contained *aac(6′)-Ib* and *ere(A)*, encoding for erythromycin resistance (Figure 6A). pMNBM065-2 and pMNBM072 had a complex class 1 integron generated by an IS*CR1* element. These class 1 integrons contained the gene cassettes *aac(6′)-IIc* and *aadA1* in pMNBM065-2, and *aac(6′)-Ib-cr*, *arr-3*, *dfrA27* and *aadA16* in pMNBM072. In contrast, *catA* and *qnrB6* were likely acquired through the recombination involving the IS*CR1* element-class 1 integron 3′-CS fusion variants, respectively (Figure 6A). The four variants of the class 1 integron gene cassette promoter located in *intI1* gene (Pc)—PcW, PcH1, PcH2 and PcS—are known to differ in transcriptional strengths: PcW (ancestral and the weakest form), PcS (the strongest form), PcH1 (stronger than PcW but weaker than PcH2) and PcH2 (between PcS and PcH1) [33,34,35]. Two kinds of promoters, PcW and PcH1, of the class 1 integrons were found in the captured plasmids (Figure 6B). The promoters in pMNBM065-2 and pYKBL037 exhibited higher predicted strength than those in pYKCS045, pMNBM072, pMNBL073 and pMNBL056 (Figure 6B).

Collectively, our findings highlight two major patterns among the captured plasmids. First, IncN and IncC plasmids exhibited high structural similarity, including conserved accessory resistance regions, to clinically derived plasmids reported from geographically distant regions, indicating that clinically associated plasmids are also present in the urban river environment. Second, plasmids such as the multi-replicon IncU+IncQ2γ plasmid and several IncP plasmids displayed accessory-region architectures characteristic of environmental plasmids, suggesting that urban rivers can also serve as sites where potentially novel MDR plasmids persist and diversify. Taken together, these results support the idea that urban rivers, such as the Tama River, can function as ecological hubs where clinically derived and environmentally adapted resistance plasmids coexist, interact, and potentially disseminate. However, the extent to which these interactions translate into plasmid dissemination, their abundance in situ, and their broader epidemiological significance cannot be directly inferred from the present study, as described below.

This study has several limitations that should be acknowledged. First, the plasmids analyzed here were obtained using an exogenous plasmid capture approach, which selectively enriches plasmids that are transferable under the applied experimental conditions. Consequently, this method does not allow quantitative estimation of the absolute abundance or copy numbers of specific plasmids or antimicrobial resistance genes in the original river sediment samples. Second, only a subset of plasmids identified in our previous study was subjected to detailed analysis, and the 11 ARG-bearing plasmids examined here may not fully represent the overall plasmid diversity present in the Tama River. In addition, antimicrobial susceptibility testing and conjugation assays were conducted using selected laboratory and environmental host strains, which may not reflect the full range of host–plasmid interactions occurring in situ. Finally, sampling was conducted at limited time points, and seasonal or temporal variations in microbial community composition and plasmid dynamics were not assessed. Future studies integrating culture-independent quantitative approaches, such as metagenomic analyses, together with broader temporal sampling will be essential to further elucidate the abundance, diversity, and dissemination dynamics of resistance plasmids in urban river environments.

## 3. Materials and Methods

### 3.1. Bacterial Strains, Plasmids, and Culture Conditions

*Escherichia coli* MG1655RGFP is a rifampicin-resistant derivative of *E. coli* MG1655, in which a mini-Tn*5*-Km-P_A1/O4/O3_-RBSII-gfpmut3* cassette was chromosomally inserted at position 578,584 nt (NC_000913), conferring kanamycin and chloramphenicol resistance [34]. *E. coli* DH5α was used as a standard laboratory strain for plasmid propagation and maintenance. *Metapseudomonas resinovorans* CA10dm4RGFP (former *Pseudomonas resinovorans*) is a rifampicin-resistant strain, in which a miniTn*7*(Gm)-P_A1/O4/O3_-*gfp*-a cassette was integrated into the chromosome immediately downstream of the *glmS* gene (position 6,265,580 nt, NC_021499), conferring gentamicin and chloramphenicol resistance [36]. *E. coli* and *Metapseudomonas resinovorans* strains were cultivated in Luria broth (LB) [37] at 30 °C or 37 °C with shaking at 180 rpm. R2A plates containing 1.5% agar were used for filter matings. Ampicillin (Ap, 50 μg/mL), kanamycin (Km, 30 μg/mL for plasmid capture and 50 μg/mL for the other experiments), gentamicin (Gm, 30 μg/mL), and rifampicin (Rif, 30 μg/mL for plasmid capture and 50 μg/mL for the others) were added to the medium. Cycloheximide (100 μg/mL) was added to prevent fungal growth. For plate cultures, LB was solidified using 1.5% agar (wt/vol). The broad-host-range plasmid pBBR1MCS-2 [38], carrying a kanamycin resistance marker and a mobilization (*mob*) region compatible with IncP, IncQ, and IncW plasmids, was used where appropriate.

### 3.2. Collection of Environmental Samples and Exogenous Plasmid Captures

River sediment samples were collected using sterile spatulas and transferred into sterile containers from six sites located in the upstream, midstream and downstream regions of the Tama River in Tokyo, Japan in 6 July 2019, 6 October 2019, and 15 February 2020 (Figure 1). The sampling sites were as follows: Tama1 (35.803872 N 139.194108 E), Tama2 (35.776822 N 139.287567 E), Tama3 (35.695053 N 139.363358 E), Tama4 (35.652894 N 139.504669 E), Tama5 (35.601667 N 139.624847 E), and Tama6 (35.544467 N 139.725906 E). Samples were transported to the laboratory within a few days at room temperature and subsequently used for exogenous plasmid capture experiments, as described previously [10]. In brief, exogenous plasmid capture was performed using GFP-tagged *Escherichia coli* MG1655RGFP or *Metapseudomonas resinovorans* CA10dm4RGFP as recipient strains. Environmental samples were mixed with recipient cells on membrane filters and incubated to allow conjugative transfer (filter mating). Biparental mating enabled direct capture of self-transmissible tetracycline-resistant plasmids from environmental bacteria, whereas triparental mating employed an intermediate donor strain to facilitate mobilization of non-self-transmissible plasmids by helper plasmids (pBBR1MCS-2). Transconjugants were selected on antibiotic-containing media and screened based on GFP fluorescence.

### 3.3. Antibiotic Resistance Testing

Antibiotic resistance testing for the host of plasmids pMNDW109 and pMNDX110 was performed. For this testing, ampicillin (Ap, 50 µg/mL), gentamicin (Gm, 30 µg/mL), and tetracycline (Tc, 12.5 µg/mL) were added to LB. Resistance testing for the other plasmids had already been performed in our previous report [10]. The antibiotics were chosen to correspond directly to the antimicrobial resistance genes identified on the analyzed plasmids, allowing targeted phenotypic assessment of plasmid-encoded resistance determinants. The qualitative resistance (R/S) was assessed based on growth or no growth in liquid LB medium supplemented with antibiotics.

For pYKCS045, the determination of minimum inhibitory concentrations (MICs) was additionally performed using the broth microdilution method with Mueller Hinton Broth, following the Clinical and Laboratory Standards Institute CLSI M07 protocol (three biological replicates) [39]. This assay was performed in two independent experiments, and the same MIC value was observed in all replicates.

### 3.4. Plasmid Sequencing and Annotation

The nucleotide sequences of pMNDW109 and pMNDX110 were determined using MiSeq platform (Illumina, San Diego, CA, USA). 151 bp paired-end libraries were prepared using the Nextera XT DNA Library Preparation Kit (Illumina). Assembly of the sequencing reads was performed using SPAdes v. 3.14.1 [40] with default parameters. In addition, PCR was performed to confirm the connection between sequence fragments. These plasmids were annotated using DFAST (https://dfast.ddbj.nig.ac.jp/, accessed on 29 August 2025) [41] and their accession numbers in DDBJ are LC895901.1 and LC895902.1 (listed in Table 1). Other plasmids were previously determined and annotated [10]; their accession numbers deposited in DDBJ are shown in Table 1. ARGs were detected using ResFinder database (https://bitbucket.org/genomicepidemiology/resfinder_db/src/master/, accessed on 9 August 2025). Plasmid maps were visualized using Easyfig v.2.2.5 [42]. The structures of plasmids were compared using Easyfig.

## Figures and Tables

**Table 1:** Antimicrobial resistance plasmids obtained from the Tama River.

Plasmid	Inc_Group	Size (bp)	Source	Methods *	Accessory Genes	Accession Number
pMNBM065-1+	PromAγ	41,504	Tama5	B	no accessory genes	LC623891.1
pMNBM065-2	IncU, IncQ2γ	67,638	Tama5	B	*tetRC*, IS*6100*, class 1 integron [*aac(6′)-IIc*, *aadA1*, *qacEΔ1*-*sul1*]; IS*CR1* element; *catA*, *qacEΔ1*-*sul1* (repeat unit x6), *orf5*	LC895900.1
pMNBM072	IncN	55,403	Tama5	B	Class 1 integron [*aac(6′)-Ib-cr*, *arr-3*, *dfrA27*, *aadA16*, *qacEΔ1*-*sul1*, IS*CR1* element *qnrB6*, *qacEΔ1*-*sul1*-*orf5*], *tetAR*	LC663726.1
pMNBL073	IncC	149,764	Tama4	B	Class 1 integron (*aadA2*, *qacEΔ1*, *sul1*), *dfrA23*, *aph(4)-Ia*, *aac(3)-IVa*, *floR*, *tetAR*, *strAB*, *sul2*	LC663722.1
pYKCS045+	IncPγ	49,370	Tama5	T	Tn*402*-like class 1 integron [*bla*_GES-24_, *aac(6′)-Ib4*]	LC623929.1
pMNBL056+	IncPε	52,432	Tama4	B	Class 1 integron (*dfrB1*, *qacEΔ1*-*sul1*-*orf5*, *tetRA*)	LC623890.1
pYKBL037+	IncPι	64,506	Tama4	T	Transposon (*strAB*, class 1 integron [*aac(6′)-Ib*, *ere(A)*, *qacEΔ1*-*sul1*-*orf5*], transposon (*blaA*, *qac*)	LC623919.1
pMNDW109	IncPβ-1	98,131	Tama5	B	Tn*3*-family transposon (*mer* operon, IS*1071*, *dmfR*, IS*1071*), IS*6100*, *sul1*, *qacEΔ1*, *aadA2*, IS*6* composite transposon (*mphE, msrE*, IS*26*, *tetRC*, IS*26*, *tetRC*), *blaD*	LC895901.1
pMNDX110	IncPβ-1	77,896	Tama6	B	*CusA/CzcA*, *HlyD*, *heavy metal resistance*, *blaC*, IS*26*, *msrE*, *mphE*, IS*26*, *tetCR*, IS*26*	LC895902.1
pMNBM077+	IncPκ	53,339	Tama5	B	Transposon (*tetAR*, transposon)	LC623892.1
pYKTC011-1+	IncPβ-1	57,620	Tama6	T	Tn*501*(remnant)(*mer* operon), IS*21*, Tn*3*-family (*bla*_NPS_), *relE*	LC623931.1
pMNBL076-1	Not classified	16,094	Tama4	B	*tetA*	LC663723.1

* T, plasmid capture by triparental matings; B, capture by biparental matings. +: Genomic information of these plasmids were previously reported [10].

**Table 2:** Antibiotic resistance and sensitivity of the hosts. (R, resistant; S, sensitive) *.

Plasmid	Inc_Group	Tc(12.5)	Gm(30)	Ap(50)	Km(50)	Cm(30)	Sm(25)	Sm(50)	Em(25)
pMNBM065-2	IncU, IncQ2γ	R	-	-	-	R	-	-	-
pMNBM072	IncN	R	S	R	R	-	-	R	-
pMNBL073	IncC	R	R	S	S	R	-	-	-
pYKCS045+	IncPγ	-	S	R	S	-	-	-	-
pMNBL056+	IncPε	R	S	S	-	-	-	-	-
pYKBL037+	IncPι	-	S	R	R	-	R	S	R
pMNDW109	IncPβ-1	R	S	R	-	-	-	-	-
pMNDX110	IncPβ-1	R	S	S	-	-	-	-	-
pMNBM077+	IncPκ	R	-	S	-	-	-	-	-
pYKTC011-1+	IncPβ-1	-	-	R	-	-	-	-	-
pMNBL076-1	Not classified	-	-	-	-	-	-	-	-

* Tc, tetracycline; Gm, gentamicin; Ap, ampicillin; Km, kanamycin; Cm, chloramphenicol; Sm, streptomycin; Em, erythromycin. “-“ indicates not tested. +: The antimicrogram of these plasmids were previously reported [10].

## Data Availability

The complete nucleotide sequences of plasmids analyzed in this study have been deposited in the DDBJ database under the accession numbers LC895901.1 and LC895902.1. The other accession numbers previously deposited are listed in Table 1.

## Abbreviations

The following abbreviations are used in this manuscript:

ARG	Antimicrobial resistance gene
Cm	Chloramphenicol
CS	Conserved segment
DDBJ	DNA Data Bank of Japan
ENA	European Nucleotide Archive
GFP	Green fluorescent protein
IS	Insertion sequence
ISCR	Insertion sequence common region
kb	Kilobase(s)
MGE	Mobile genetic element
MIC	Minimum inhibitory concentration
oriIS	Origin of insertion sequence replication
PCR	Polymerase chain reaction
PLSDB	Plasmid database
Rep	Replication protein
RND	Resistance–nodulation–cell division
Tn	Transposon
